# Cost-Effectiveness of Antiobesity Drugs for Adolescents With Severe Obesity

**DOI:** 10.1001/jamanetworkopen.2023.36400

**Published:** 2023-10-12

**Authors:** Shweta Mital, Hai V. Nguyen

**Affiliations:** 1 College of Pharmacy, University of Manitoba, Winnipeg, Manitoba, Canada; 2 School of Pharmacy, Memorial University of Newfoundland, St John’s, Newfoundland and Labrador, Canada

## Abstract

**Importance:**

Although the American Academy of Pediatrics has recommended treatment with antiobesity drugs for adolescents, the cost-effectiveness of antiobesity drugs for this population is still unknown.

**Objective:**

To quantify cost-effectiveness of different antiobesity drugs available for pediatric use.

**Design, Setting, and Participants:**

This economic evaluation used a Markov microsimulation model with health states defined by obesity levels. Effectiveness was measured by quality-adjusted life-years (QALYs) and costs were calculated from third-party payer perspective, estimated in 2023 US dollars over a 10-year horizon. Data were obtained from the published literature.

**Intervention:**

Antiobesity drugs orlistat, liraglutide, semaglutide, and phentermine-topiramate vs no treatment. Metformin hydrochloride and 2 types of bariatric surgical procedures (sleeve gastrectomy and gastric bypass) were considered in sensitivity analysis.

**Main Outcomes and Measures:**

Incremental cost-effectiveness ratio.

**Results:**

Among the 4 antiobesity drugs currently approved for pediatric use, phentermine-topiramate was the most cost-effective with an incremental cost-effectiveness ratio of $93 620 per QALY relative to no treatment in this simulated cohort of 10 000 adolescents aged 12 to 17 years (mode, 15 years) with severe obesity (62% female). While semaglutide offered more QALYs than phentermine-topiramate, its higher cost resulted in an incremental cost-effectiveness ratio ($1 079 480/QALY) that exceeded the commonly used willingness-to-pay threshold of $100 000 to $150 000/QALY. Orlistat and liraglutide cost more and were less effective than phentermine-topiramate and semaglutide, respectively. Sleeve gastrectomy and gastric bypass were more effective than phentermine-topiramate but were also more costly, rendering them not cost-effective compared with phentermine-topiramate at the willingness-to-pay threshold of $100 000 to $150 000/QALY.

**Conclusions and Relevance:**

In this economic evaluation of weight loss drugs for adolescents with severe obesity, we found phentermine-topiramate to be a cost-effective treatment at a willingness-to-pay threshold of $100 000 to $150 000/QALY. Further research is needed to determine long-term drug efficacy and how long adolescents continue treatment.

## Key Points

**Question:**

Are antiobesity drugs currently approved for pediatric use cost-effective for treating adolescents with severe obesity?

**Findings:**

In this economic evaluation using a microsimulation model, phentermine-topiramate was found to be the most cost-effective treatment. While semaglutide could generate greater weight loss than phentermine-topiramate, its incremental cost per quality-adjusted life-year gained exceeded the commonly used willingness-to-pay threshold.

**Meaning:**

The findings indicate that among the 4 antiobesity drugs currently available for pediatric use, phentermine-topiramate was the most cost-effective option to treat adolescents with severe obesity.

## Teaser

This economic evaluation assesses the cost-effectiveness of pediatric antiobesity drugs.

## Introduction

Childhood obesity increases the risk of developing adult obesity and reduces social and emotional competence among adolescents.^1,2^ It is projected that more than half of today’s youth in the US will develop obesity by age 35 years.^2^ To tackle this growing challenge, the American Academy of Pediatrics (AAP) released new guidelines in January 2023 recommending treatment with antiobesity drugs for adolescents aged 12 years and older and bariatric surgery for those aged 13 years and older.^3^ The goal is early and intensive treatment rather than watchful waiting for children to outgrow obesity.^4^ Following the AAP guidelines, a similar review and update of guidelines to treat childhood obesity has been under way in Canada.^5^

The AAP guidelines came soon after the US Food and Drug Administration approved 2 new antiobesity drugs for children aged 12 years and older: semaglutide (a glucagon-like peptide [GLP]−1 receptor agonist) and extended-release phentermine-topiramate (a combination of a sympathomimetic amines and an anticonvulsant) in addition to the previously approved liraglutide (a GLP-1 agonist) and orlistat (a gastrointestinal lipase inhibitor). With obesity experts hailing semaglutide as a game changer and celebrities and social media popularizing antiobesity drugs, demand for these drugs has surged in recent months.^6,7,8^ In October 2022, semaglutide (sold under trade names of Wegovy and Ozempic) was declared in short supply in the US.^9^ The Canadian province of British Columbia was forced to ban sales of Ozempic to non-Canadians as the US turned to buying the drug in Canada.^10^

Despite the enormous public interest in and the potential downstream health benefits associated with these drugs, concerns regarding economic value and treatment access remain. While prices of orlistat and phentermine-topiramate range from around $1500 to $8500 per year, other drugs—such as semaglutide and liraglutide—cost more than $12 000 per year,^11^ which is well above the minimum prices for these drugs to be profitable with a 10% profit margin.^12^ Furthermore, even as clinical trials indicate that these drugs are effective, the efficacy varies considerably across drugs, with a 2 to 17 percentage point reduction in body mass index (BMI) after 1 year relative to placebo.^13,14,15,16^ Their long-term efficacy and the prospect of weight regain after patients stop treatment are also unknown. Consequently, except for a few Medicaid programs, most insurance plans in the US do not currently cover antiobesity drugs.^17^

Previous studies have assessed the cost-effectiveness of antiobesity drug treatment among adults.^18,19,20,21,22,23^ Despite some variations in types of interventions compared, assumptions around long-term treatment efficacy, and findings as to which drug was the most cost-effective, they broadly found antiobesity drug treatment among adults to be cost-effective. However, the cost-effectiveness of these drugs for obesity treatment among adults may not apply to the context of adolescents, which differs from the adult population in terms of both treatment efficacy and likelihood of adverse effects.^24^ This modeling study aims to provide a cost-effectiveness analysis of antiobesity drugs for adolescents to inform treatment and reimbursement decisions for this population.

## Methods

This was an economic evaluation modeling study that did not involve human participants and relied exclusively on data from publicly available published literature. Hence, ethics approval was not required as per Newfoundland and Labrador Health Research Ethics Board guidelines. This study followed the Consolidated Health Economic Evaluation Reporting Standards (CHEERS) reporting guideline.^25^ While patients or other stakeholder groups were not directly involved, our study was designed to account for patient and stakeholder concerns and debates noted in media reports.

### Treatments Under Comparison

Our comparison included 4 antiobesity drugs approved for pediatric use in the US—orlistat, liraglutide, semaglutide, and extended-release phentermine-topiramate—and no drug treatment. Details of strength and route of administration of these drugs are provided in the eMethods in Supplement 1.

In sensitivity analyses, we additionally compared off-label extended-release metformin hydrochloride and 2 types of bariatric surgical procedures: gastric bypass and sleeve gastrectomy. While bariatric surgery can generate larger weight loss than drugs, it entails high upfront costs, risk of serious complications, and the possibility of weight regain after surgery.

### Study Cohort and Model Structure

We developed a Markov microsimulation model to estimate and compare the costs and quality-adjusted life-years (QALYs) of the different treatments. The model simulated 10 000 adolescents aged 12 to 17 years (mode, 15 years) and with BMI (calculated as weight in kilograms divided by height in meters squared) greater than 120% of the 95th percentile of BMI by age and sex (36 on average); 62% of adolescents were female. These characteristics were similar to those of participants in the randomized clinical trials for the 4 weight loss drugs^13,14,15,16^ (eTable 1 in Supplement 1).

**Figure 1.**
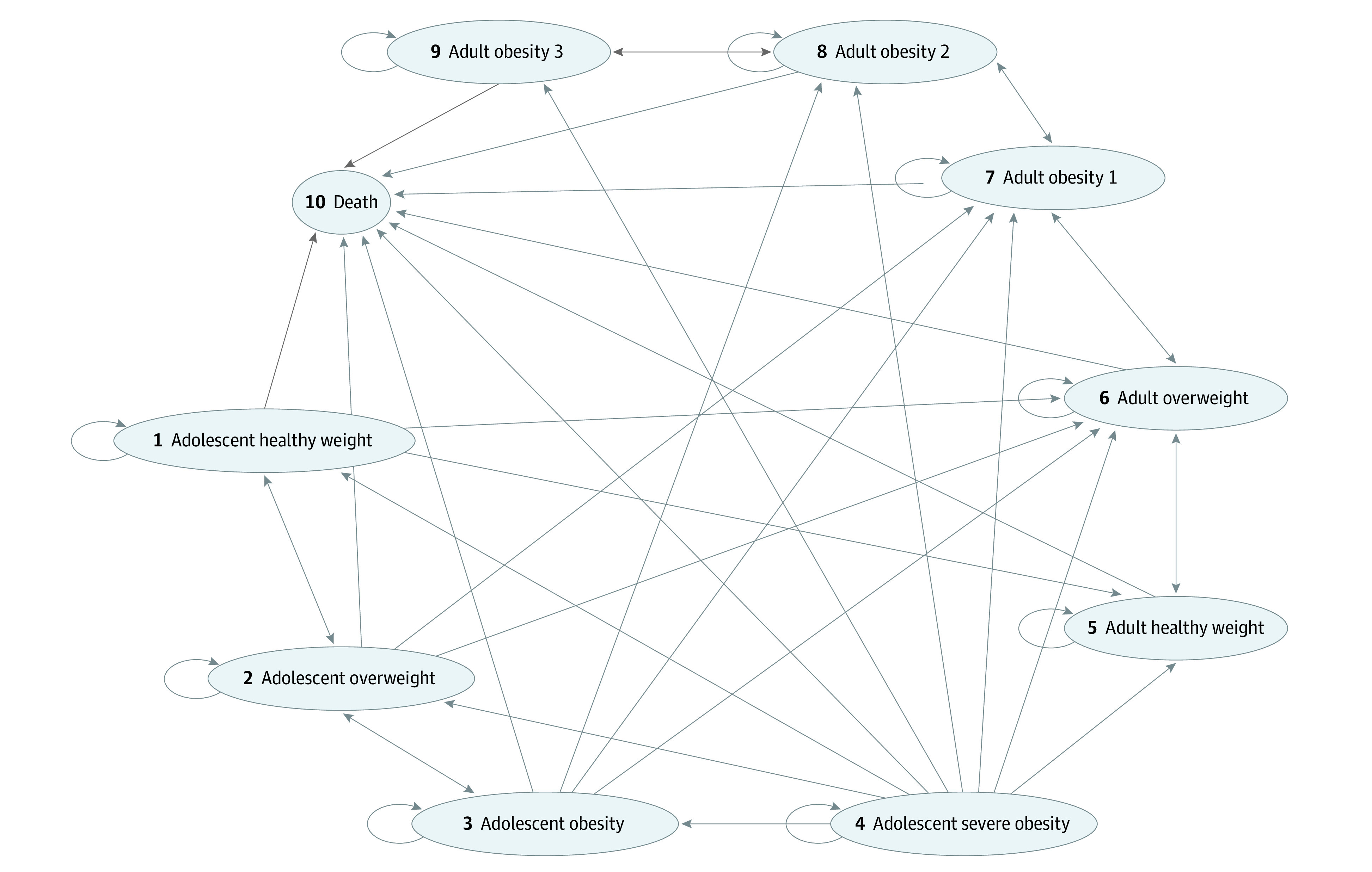
Transition Across Body Mass Index (BMI) States Adolescent healthy weight indicates BMI (calculated as weight in kilograms divided by height in meters squared) between 5th and 85th percentile; adolescent overweight, BMI between 85th and 95th percentile; adolescent obesity, BMI above 95th percentile but below 120% of the 95th percentile or 35; adolescent severe obesity, BMI above 120% of the 95th percentile or 35; adult healthy weight, BMI less than 25; adult overweight, BMI 25 to 30; adult obesity 1, BMI 30 to 35; adult obesity 2, BMI 35 to 40; and adult obesity 3, BMI 40 and higher.

The model simulated an adolescent’s weight transition across 10 health states defined by BMI and age (Figure 1). Nine of these health states corresponded to BMI categories for childhood and adult obesity and are described in the eMethods in Supplement 1. The last health state was death. All adolescents entered the model in the severe obesity state (BMI above 120% of the 95th percentile or 35) and received treatment with 1 of the weight loss drugs (depending on strategy) or no treatment.

The analysis was conducted from the third-party payer perspective. Cycle length was set at 1 year, and the time horizon was 10 years. This time horizon was chosen as it was long enough to capture the benefits of weight loss during adolescence that may extend into early adulthood. At the same time, it allowed the model to remain realistic, as individuals’ behaviors and preferences regarding weight management may change during adulthood.

### Model Inputs

#### Transitions Across Health States and Weight Loss Effects

Transitions across BMI states were determined by the extent of weight loss or gain among the treatment and placebo arms of randomized clinical trials for the 4 weight loss drugs.^13,14,15,16^ We chose trials that were conducted among US adolescents with a trial duration of at least 52 weeks. To our knowledge, only 1 trial exists for each of the 4 drugs, but samples in these trials were similar (eTable 1 in Supplement 1).

For adolescents undergoing treatment with antiobesity drugs, BMI change in year 1 was calculated by multiplying the baseline BMI with drug efficacy in the first year of treatment sourced from the clinical trial for each drug. As only 1-year efficacy data were available for each drug, we assumed that the BMI level achieved at the end of year 1 was maintained beyond that as long as the individual continued treatment.^18^ We varied this assumption in sensitivity analyses.

For adolescents in the no treatment strategy, BMI increase during year 1 followed that observed in the placebo arm of the drug trials. As trials for each of the 4 drugs had a separate placebo arm, we pooled together rates of BMI increases across the 4 trials into an average rate of BMI increase using the DerSimonian and Laird random-effects procedure.^26^ As only data on BMI increases in year 1 were available from the trials, we assumed that the estimated average rate of BMI increase in year 1 (0.96%) remained the same in each year throughout adolescence (ie, until age 20 years). When individuals entered young adulthood (ie, ages 20-25 years), we assumed that BMI increased at the rate of 0.2 per year observed for people with class 2 or 3 obesity.^27^

#### Adverse Events, Treatment Discontinuation, and Mortality Risks

All 4 drugs had similar adverse events in the trials, primarily gastrointestinal. As these events were mild or moderate, we did not account for their impact on health outcomes and health care utilization. However, our model allowed for treatment discontinuation due to adverse events (and other reasons). Probability of treatment discontinuation was based on proportion of participants who discontinued treatment in the clinical trials. As discontinuation occurred within the first year itself, we assumed that adolescents who discontinued treatment experienced no reductions in BMI and that their BMI progression followed that of adolescents in the no treatment strategy.

In each cycle, individuals faced risk of all-cause mortality. Risk of mortality was specific to age and sex and was obtained from US life tables.^28^ We assumed that adolescents did not face additional mortality risks due to obesity. However, for adults aged 20 years and older, we adjusted the age-specific mortality risks with BMI-specific hazard ratios that varied by age.^29^

#### Costs

Costs were estimated from the health system perspective. For each strategy, costs included cost of the drug, treatment-related follow-up visits, and annual general health care costs specific to BMI states (eTable 2 in Supplement 1). As antiobesity drugs are intended for long-term weight management, in the base case analysis, we assumed that individuals continued using the drug over the entire 10-year time horizon and varied this assumption in sensitivity analyses. Follow-up costs involved 2 physician visits per year as long as the individual continued using the drug. Each individual also incurred average annual health care costs specific to their BMI health state (eMethods in Supplement 1), obtained from the published literature.^30^ All costs were estimated in 2023 US dollars and discounted at 3% per year.^31^

#### Effectiveness

Effectiveness was measured by QALYs, which captured an individual’s length of life weighted by their health-related quality of life (or utility) (eTable 2 in Supplement 1). Utility was specific to age and BMI.^32,33^ All utility values were discounted at 3% per year.^31^

### Statistical Analysis

We estimated the incremental cost effectiveness ratio (ICER), calculated as the difference between the total costs of 2 strategies divided by the difference between the total QALYs. The ICER was then compared with the willingness-to-pay threshold of $100 000 to $150 000 per QALY.^34^

In addition to 1-way sensitivity analyses and probabilistic sensitivity analyses to address parameter uncertainty, we conducted several sensitivity analyses, including alternative time horizons, alternative scenarios for treatment discontinuation and long-term drug efficacy and use of real-world BMI progression rates for adolescents not receiving treatment. Moreover, as AAP guidelines recommend bariatric surgery for adolescents with severe obesity and metformin is commonly used off-label to treat pediatric obesity,^35^ we conducted analyses to include these treatments as additional comparators. Details of these analyses are provided in the eMethods in Supplement 1. The analysis was conducted using TreeAge Pro version 2023 R1.2 (TreeAge Software).

## Results

### Base Case Analysis

Among the 4 antiobesity drugs considered, phentermine-topiramate was the most cost-effective (Table 1). Orlistat and liraglutide were dominated (ie, cost more and were less effective) by phentermine-topiramate and semaglutide, respectively. Phentermine-topiramate cost $6921 more than no treatment and generated 0.07 additional QALYs, yielding an ICER of $93 620/QALY. Semaglutide was the most effective of all treatments (generating 0.08 additional QALYs compared with phentermine-topiramate). However, total cost of semaglutide treatment was $84 649 more than that with phentermine-topiramate, with a resulting ICER of $1 079 480/QALY, which exceeds the willingness-to-pay threshold of $100 000 to $150 000 per QALY. Except for phentermine-topiramate, the ICERs for each of the other drugs (vs no treatment) ranged between $600 000 and $1.3 million/QALY, indicating that these drugs were not cost-effective compared with no treatment.

**Table 1.** Base Case Cost-Effectiveness Results^a^

Strategy	Cost, $^b^	Incremental cost, $^b^	Effectiveness, QALY	Incremental effectiveness, QALY	ICER, $/QALY^b^
Comparison across all strategies^c^					
No treatment^d^	48 239	NA	7.69	NA	NA
Phentermine-topiramate	55 160	6921	7.77	0.07	93 618
Orlistat	98 137	42 977	7.75	−0.02	−1 868 154 (dominated)
Semaglutide	139 809	84 649	7.85	0.08	1 079 476
Liraglutide	157 847	18 038	7.78	−0.07	−257 660 (dominated)
Comparison vs no treatment					
Phentermine-topiramate	55 160	6921	7.77	0.07	93 618
Orlistat	98 137	49 898	7.75	0.05	979 843
Semaglutide	139 809	91 570	7.85	0.15	601 066
Liraglutide	157 847	109 608	7.78	0.08	1 331 189

Abbreviations: ICER, incremental cost-effectiveness ratio; NA, not applicable; QALY, quality-adjusted life-years.

^a^ Analyses conducted over a 10-year horizon.

^b^ All costs are in 2023 US dollars.

^c^ Each strategy compared with the next less costly strategy (excluding any dominated strategies).

^d^ Body mass index progression for the no treatment strategy is based on meta-analytic estimates from the placebo arms of randomized clinical trial data for each of the 4 drugs.^13,14,15,16^

### Sensitivity and Scenario Analyses

Sensitivity analyses considering alternative time horizons indicated that for time horizons longer than 7.6 years, the ICER for phentermine-topiramate vs no treatment would remain below the $150 000/QALY threshold (eFigure 1 in Supplement 1). Furthermore, if individuals continued treatment over a period as long as 30 years (and assuming treatment prices would eventually decline during this period), cost-effectiveness of phentermine-topiramate would improve with an ICER as low as $30 100/QALY relative to no treatment (Table 2). Meanwhile, over a 10-year time horizon, treatment duration of at least 5 years would be required for phentermine-topiramate to be cost-effective (vs no treatment) at the $150 000/QALY threshold (eFigure 2 in Supplement 1).

**Table 2.** Sensitivity Analyses

Strategy	Cost, $^a^	Incremental cost, $^a^	Effectiveness, QALY	Incremental Effectiveness, QALY	ICER, $/QALY^a^
30-y Horizon^b^					
No treatment	142 953	NA	16.66	NA	NA
Phentermine-topiramate	152 115	9162	16.97	0.30	30 097
Semaglutide	289 577	137 462	17.34	0.37	370 928
Using real-world BMI progression rate for no treatment from year 2 onward^b^					
No treatment	48 261	NA	7.63	NA	NA
Phentermine-topiramate	55 163	6902	7.74	0.12	59 893
Semaglutide	139 810	84 647	7.84	0.10	852 608
Weight loss assumed to continue beyond year 1 and up to year 5^b^					
No treatment	48 239	NA	7.69	NA	NA
Phentermine-topiramate	52 974	4735	7.79	0.09	52 325
Semaglutide	135 764	82 790	7.95	0.16	503 043
Including bariatric surgery					
No treatment	48 239	NA	7.69	NA	NA
Phentermine-topiramate	55 160	6921	7.77	0.07	93 618
Sleeve gastrectomy	77 394	22 234	7.82	0.05	469 056
Gastric bypass	87 956	10 562	7.81	−0.01	−1 623 074 (dominated)
Orlistat	98 137	20 743	7.75	−0.07	−294 624 (dominated)
Semaglutide	139 809	62 415	7.85	0.03	2 012 370
Liraglutide	157 847	18 038	7.78	−0.07	−257 660 (dominated)
Including metformin^b^					
No treatment	48 239	NA	7.69	NA	NA
Metformin	52 254	4016	7.76	0.06	64 090
Phentermine-topiramate	55 160	2905	7.77	0.01	257 747
Semaglutide	139 809	84 649	7.85	0.08	1 079 476

Abbreviations: BMI, body mass index; ICER, incremental cost-effectiveness ratio; NA, not applicable; QALY, quality-adjusted life-years.

^a^ All costs are in 2023 US dollars.

^b^ Dominated strategies are excluded.

We obtained results similar to the base case analysis when we used alternative BMI progression rates for adolescents not receiving drug treatment. When we assumed that weight loss effects of drugs continued beyond 1 year, the ICERs for both phentermine-topiramate and semaglutide were much lower than in the base case analysis ($52 320/QALY and $503 040/QALY, respectively), but the ICER for semaglutide vs phentermine-topiramate ($503 040/QALY) still exceeded the $150 000/QALY threshold.

While bariatric surgery generated higher QALYs than phentermine-topiramate, it was also more costly. The ICER for sleeve gastrectomy vs phentermine-topiramate was $469 060/QALY, which exceeded the $100 000 to $150 000/QALY threshold. Gastric bypass was dominated by sleeve gastrectomy.

Phentermine-topiramate was no longer cost-effective when we considered metformin as a comparator. Metformin was less costly and less effective than phentermine-topiramate with an ICER of $64 090/QALY relative to no treatment; however, the additional QALYs gained with phentermine-topiramate vs metformin did not justify its higher costs.

**Figure 2.**
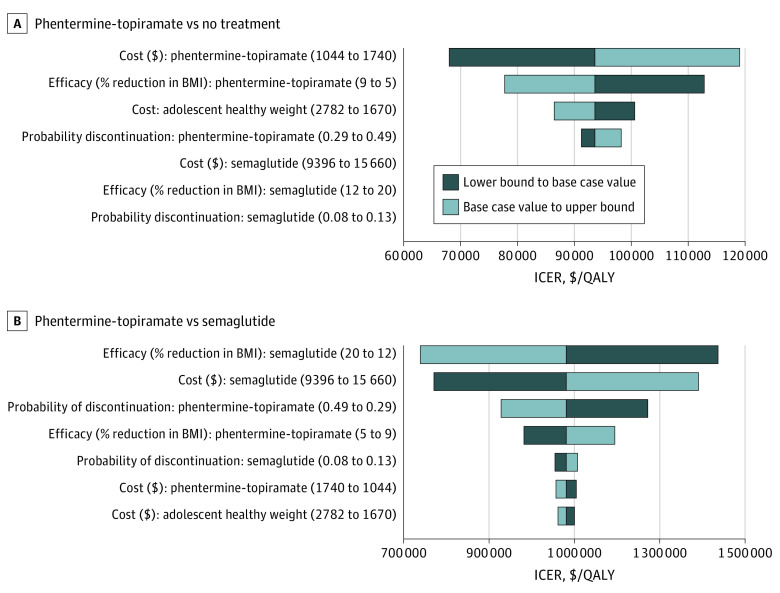
Sensitivity of Incremental Cost-Effectiveness Ratios to Model Inputs BMI indicates body mass index; ICER, incremental cost-effectiveness ratio; QALY, quality-adjusted life-years.

Tornado diagrams (Figure 2) show that the ICER was most sensitive to drug costs and efficacy. However, for all values of key parameters in the range of ±25% of their base case values, the ICER for phentermine-topiramate vs no treatment was below the $150 000/QALY threshold while that for semaglutide vs phentermine-topiramate exceeded the $100 000 to $150 000/QALY threshold. Cost-effectiveness acceptability curves from the probabilistic sensitivity analyses indicated that phentermine-topiramate would be cost-effective in 56% and 92% of all iterations at willingness-to-pay thresholds of $100 000/QALY and $150 000/QALY, respectively (eFigure 3 in Supplement 1).

## Discussion

The findings in this economic evaluation suggest that phentermine-topiramate was the most cost-effective of all antiobesity drugs approved for the pediatric population. This finding arises as phentermine-topiramate costs the least while generating modest weight loss. Semaglutide was more effective than phentermine-topiramate in terms of weight loss but it was also more expensive, such that the additional clinical benefits did not justify the additional costs.

Sensitivity analyses suggest that cost-effectiveness of antiobesity drugs may improve if benefits of these drugs accrue for sufficiently long time periods. To the extent that we considered a 10-year time horizon, our cost-effectiveness estimates may therefore be conservative if benefits of these drugs extend into adulthood. Previous studies examining cost-effectiveness of antiobesity drugs among adults find phentermine-topiramate treatment to be cost-effective over a lifetime horizon with ICER as low as $8000 per QALY.^18^

While we found that metformin compares favorably with other antiobesity drugs in terms of cost-effectiveness, it is not approved by the US Food and Drug Administration for weight loss. Moreover, its mechanism of action for weight loss remains unclear.^36^

Our finding that bariatric surgery is not cost-effective vs antiobesity drugs for adolescents stands in contrast with previous findings in studies with adult populations. A number of factors could explain this difference. Studies for adults have only compared bariatric surgery against orlistat and semaglutide (while our study also included phentermine-topiramate and liraglutide). Also, health care cost reductions and utility benefits of weight loss are likely to be larger (due to greater risk of obesity-related complications) in adulthood than in adolescence, giving rise to the cost-effectiveness of bariatric surgery for adults. Our finding for adolescents is significant given AAP’s recommendation of bariatric surgery treatment for adolescents with severe obesity. Yet we urge caution in interpreting these findings, as the study sample in the Teen-Longitudinal Assessment of Bariatric Surgery (Teen-LABS study)^37^ from which data on efficacy of bariatric surgery were obtained for our study, was slightly older and had higher mean baseline BMI than the study samples in the drug trials. While head-to-head trials have not directly compared bariatric surgery vs antiobesity medications treatment for adolescents, our results provide the first insights into the relative economic value of these 2 recommended antiobesity treatments for adolescents.

Our findings have implications for clinical and reimbursement decision-making. Several recent studies have argued that the large number of individuals eligible for antiobesity treatment in the US implies that coverage for expensive drugs such as GLP-1 agonists will significantly strain payers’ budgets.^12,38,39^ Our findings suggest that, as a weight-loss treatment for adolescents, the relatively low-cost phentermine-topiramate will both place smaller demands on health care budgets and offer better value for money. Yet the budgetary implications for payers can still be substantial, and identifying which demographic subgroups can gain most from these drugs will be important.^6^

### Limitations

This study has limitations. First, only 1-year weight loss effects for all drugs were available and their long-term efficacy is yet unknown. Moreover, while long-term weight maintenance requires that antiobesity drugs be used for a long time, it is not known for how long adolescents would continue drug treatment. Second, as no clinical trials (to our knowledge) have directly compared the 4 antiobesity drugs considered, efficacy of these drugs had to be sourced from different trials. Network meta-analyses were also not feasible due to the availability of only a single trial for each drug. However, we note that patient characteristics across the 4 trials were very similar. Furthermore, we obtained similar conclusions when we used real-world BMI progression rates for individuals not receiving treatment. Third, there are safety concerns that semaglutide and liraglutide may increase risk of thyroid cancer and that phentermine-topiramate may lead to birth defects. While these outcomes were not observed within clinical trials and were thus not included in our model, they could tip the balance of cost-effectiveness. Fourth, semaglutide and liraglutide as GLP-1 agonists can help improve diabetes outcomes. While these benefits are less relevant for adolescents than for adults and our model did not capture them explicitly, these benefits are indirectly captured through changes in annual health care costs and utilities associated with higher BMI health states. Fifth, as our study relied on efficacy data from drug trials and the Teen-LABS study, generalizability of our findings may be affected by the relatively higher representation of female participants and White participants in these studies.

## Conclusions

In this economic evaluation of weight loss drugs for adolescents with severe obesity, phentermine-topiramate was found to be a cost-effective pharmaceutical intervention at conventional standards of cost-effectiveness. As data on long-term weight loss effects from more nationally representative samples become available, the analyses in this study can be adapted and updated to reassess the cost-effectiveness of these drug therapies.

## Notes

Supplement 1. **eTable 1.** Comparison of key baseline characteristics of study participants in randomized controlled trials for the four drugs
eMethods.
**eTable 2.** Model inputs **eFigure 1.** Threshold time horizon **eFigure 2.** Threshold time to discontinuation **eFigure 3.** Cost-effectiveness acceptability curve
eReferences.

Supplement 2.
Data Sharing Statement
