# Supplementary material for: Cost-Effectiveness of Antiobesity Drugs for Adolescents With Severe Obesity
Source: JAMA Netw Open. 2023 Oct 12;6(10):e2336400. doi: 10.1001/jamanetworkopen.2023.36400 (PMC10570871; doi:10.1001/jamanetworkopen.2023.36400)
Supplement: Supplement 1. — eTable 1. Comparison of key baseline characteristics of study participants in randomized controlled trials for the four drugs eMethods. eTable 2. Model inputs eFigure 1. Threshold time horizon eFigure 2. Threshold time to discontinuation eFigure 3. Cost-effectiveness acceptability curve eReferences. [file jamanetwopen-e2336400-s001.pdf]

Supplemental Online Content

Mital S, Nguyen HV. Cost-effectiveness of antiobesity drugs for adolescents with severe obesity. *JAMA Netw Open*. 2023;6(10):e2336400.
doi:10.1001/jamanetworkopen.2023.36400

eTable 1. Comparison of key baseline characteristics of study participants in randomized controlled trials for the four drugs

eMethods.

eTable 2. Model Inputs

eFigure 1. Threshold time horizon

eFigure 2. Threshold time to discontinuation

eFigure 3. Cost-effectiveness acceptability curve

This supplemental material has been provided by the authors to give readers additional information about their work.

© 2023 Mital S and Nguyen HV. *JAMA Network Open.*

eTable 1: Comparison of key baseline characteristics of study participants in randomized controlled trials for the four drugs

	Phentermine-Topiramate[1]		Orlistat[2]		Semaglutide[3]		Liraglutide[4]		Metformin[5]		Bariatric surgery[6]	
	Treated (N=56)	Placebo (N=113)	Treated (N=352)	Placebo (N=181)	Treated (N=134)	Placebo (N=67)	Treated (N=125)	Placebo (N=126)	Treated (N=39)	Placebo (N=38)	Gastric Bypass (N=161)	Sleeve Gastrectomy (N=67)
Age, mean	13.9	14.0	13.6	13.5	15.5	15.3	14.6	14.5	14.8	15.0	17	17
Female (%)	56	54	65	71	63	61	57	62	67	66	78	67
Weight, mean	108.5	102.2	97.7	95.1	109.9	102.6	99.3	102.2	95.9	101.8	151	144
BMI, mean	39.0	36.4	35.7	35.4	37.7	35.7	35.3	35.8	35.9	35.9	54	50

Note: Top dose of phentermine-topiramate considered.

© 2023 Mital S and Nguyen HV. *JAMA Network Open.*

eMethods

Details of anti-obesity drugs under comparison

Our analysis compared 4 anti-obesity drugs approved for pediatric use in the US: orlistat (120mg 3 times daily delivered orally), liraglutide (3mg daily administered subcutaneously), semaglutide (2.4mg weekly administered subcutaneously) and phentermine + topiramate extended-release (15mg/92mg daily delivered orally), and the option of no drug treatment. In line with clinical trials of anti-obesity drugs, individuals in each anti-obesity drug treatment and 'No treatment' strategy additionally received standard lifestyle therapy.

In sensitivity analyses, we additionally compared metformin hydrochloride extended-release (2000mg daily delivered orally) – a drug commonly used off-label for weight loss, and two types of bariatric surgeries, namely, gastric bypass and sleeve gastrectomy, with the above drugs and no treatment.

Health states

During adolescence, individuals could move across four health states corresponding to BMI categories for childhood obesity defined by the Centers for Disease Control and Prevention[7]: healthy weight (BMI between 5th and 85th percentile), overweight (BMI between 85th and 95th percentile), obesity (BMI above 95th percentile but below 120% of the 95th percentile or below 35 kg/m^2), and severe obesity (BMI above 120% of the 95th percentile or above 35 kg/m^2). As individuals moved into adulthood (age above 20 years), they transitioned through five states: normal weight (BMI<25kg/m^2), overweight (BMI 25-30kg/m^2), obesity 1 (BMI 30-35kg/m^2), obesity 2 (BMI 35-40kg/m^2), and obesity 3 (BMI >=40kg/m^2).

BMI-specific annual health care costs

Each individual in the model incurred average annual general health care costs specific to their age (adolescent vs adult) and BMI category in each cycle. These costs were higher for individuals with obesity than those without and implicitly captured the costs of obesity-related comorbidities (such as diabetes, hypertension or sleep apnea). Thus, if an adolescent transitioned from the 'severe obesity' to 'obesity' health state between years 1 and 2 as a result of anti-obesity treatment, they faced higher health care costs (on average) in year 1 than in year 2. Furthermore, as obesity-related comorbidities become more likely with increasing age, the average annual health care costs for each BMI health state were higher for adults than for adolescents.

Sensitivity analyses

We conducted several sensitivity analyses to examine the robustness of our results. First, we assessed how the cost-effectiveness of anti-obesity drugs varied over alternative time horizons (instead of the 10-year time horizon used in the base case analysis). We also considered a 30-year time horizon assuming that the price of each anti-obesity drug fell to its minimum possible price[8] after the first 20 years. Second,

© 2023 Mital S and Nguyen HV. *JAMA Network Open.*

we considered alternative scenarios where all adolescents who continued treatment beyond 1 year discontinued it between years 2 and 10. We assumed that after discontinuing treatment, adolescents regained their initial BMI within two years (based on trends observed after semaglutide discontinuation[9]) and BMI progression followed that of an untreated individual thereafter. Third, for adolescents who did not receive treatment, instead of assuming BMI progression rate year 2 onwards to be the same as that in year 1, we used real-world BMI progression rates. These age-specific rates – which were calculated among children or adolescents who ever had severe obesity and were enrolled in the POOL obesity registry – were slightly higher than those used in the base case analysis[10]. Fourth, we examined the sensitivity of our results to our assumption regarding long-term drug efficacy. Instead of assuming that, beyond 1 year, the BMI remains constant at the level achieved at the end of 1 year, we assumed that the BMI continues to decline up to year 5 but that the rate of decline falls by half in each successive year.

Fifth, as AAP guidelines also recommend bariatric surgery as a treatment, for adolescents with severe obesity, we expanded our analysis to include gastric bypass and sleeve gastrectomy as additional comparators. Weight loss effects of bariatric surgery were obtained from the TEEN-LABS study which prospectively studied over 200 adolescents who underwent bariatric surgery at 5 centres in the US[6]. These effects were available for 5 years for gastric bypass and 3 years for sleeve gastrectomy[6,11]. Beyond this time, we assumed that BMI was constant. Data on risk of complications, costs and utility losses associated with bariatric surgery were obtained from the published literature (eTable 2). Lastly, as, metformin is commonly used off-label for weight loss, we considered metformin as an additional treatment strategy. Weight loss effects of metformin after 52 weeks were obtained from a randomized controlled trial conducted across 6 research centres in the US[5].

© 2023 Mital S and Nguyen HV. *JAMA Network Open.*

eTable 2: Model Inputs

Variable	Value	Source
Costs (in 2023 US$)		
Drug costs (per year)		
Phentermine-topiramate	1,392	12
Orlistat	8,544	12
Semaglutide	12,528	12
Liraglutide	15,540	12
Metformin	540	13
Gastric bypass	32,092	14
Sleeve gastrectomy	30,349	14
Annual health care costs		
Healthy weight – adolescent	2,226	15
Overweight – adolescent	2,201	15
Obesity – adolescent	2,237	15
Severe obesity - adolescent	2,595	15
Normal weight - adult	4,981	15
Overweight – adult	5,719	15
Obesity 1 – adult	6,741	15
Obesity 2 - adult	8,662	15
Obesity 3 - adult	8,662	15
Cost of physician visits[a] & surgery-related complications		
Physician visit (per visit)	134	16
Dietary supplementation after bariatric surgery (per year)	116	17
Peri-procedure major complication	57,011	14
Peri-procedure minor complication	1,749	14
Post-procedure major complication	62,770	14
Post-procedure minor complication	1,096	14
Mortality hazard ratios		
Normal weight - adult	1	18
Overweight – adult	1.83 (age 20-29), 0.72 (age 30-44), 1.08 (age 45-64), 0.89 (age 65+)	18
Obesity 1 - adult	1.77 (age 20-29), 1.18 (age 30-44), 1.27 (age 45-64), 0.92 (age 65+)	18
Obesity 2 – adult	1.68 (age 20-29), 1.69 (age 30-44), 2.30 (age 45-64), 1.10 (age 65+)	18
Obesity 3 - adult	4.91 (age 20-29), 1.48 (age 30-44), 1.86 (age 45-64), 1.27 (age 65+)	18
Treatment efficacy (% BMI reduction)		
Phentermine-topiramate	7.11	1
Orlistat	1.5	2
Semaglutide	16.1	3
Liraglutide	4.29	4
Metformin	2.47	5
Gastric bypass[b]	31.4, 31, 29.5, 27.75, 26	6,11
Sleeve gastrectomy[b]	31.6, 29.9, 27.1	6

© 2023 Mital S and Nguyen HV. *JAMA Network Open.*

Variable	Value	Source
Treatment discontinuation (in 1st year), %		
Phentermine-topiramate	39	1
Orlistat	35	2
Semaglutide	10	3
Liraglutide	19	4
Probability of complications and death after surgery[c]		
Peri-procedure major complication – Gastric bypass	0.093	19
Peri-procedure major complication – Sleeve gastrectomy	0.045	19
Peri-procedure minor complication – Gastric bypass	0.168	19
Peri-procedure minor complication – Sleeve gastrectomy	0.119	19
Post-procedure major complication – Gastric bypass	0.033	20
Post-procedure major complication – Sleeve gastrectomy	0.017	20
Post-procedure minor complication – Gastric bypass	0.023	20
Post-procedure minor complication – Sleeve gastrectomy	0.022	20
Post-procedure mortality	0.019	11
Utilities		
Healthy weight – adolescent	0.98 (age <18); 0.95 (age 18-19, male); 0.93 (age 18-19, female)	21
Overweight - adolescent	0.97 (age <18); 0.94 (age 18-19, male); 0.91 (age 18-19, female)	21
Obesity – adolescent	0.95 (age <18); 0.92 (age 18-19, male); 0.88 (age 18-19, female)	21
Severe obesity – adolescent	0.95 (age <18); 0.92 (age 18-19, male); 0.88 (age 18-19, female)	21
Normal weight - adult	0.91 (age 20-30); 0.89 (age 31-40); 0.86 (age 41-50); 0.83 (age 51-60); 0.81 (age 61-70); 0.79 (age 70+)	17
Overweight - adult	0.91 (age 20-30); 0.89 (age 31-40); 0.86 (age 41-50); 0.83 (age 51-60); 0.81 (age 61-70); 0.79 (age 70+)	17
Obesity 1 – adult	0.89 (age 20-30); 0.86 (age 31-40); 0.82 (age 41-50); 0.80 (age 51-60); 0.79 (age 61-70); 0.76 (age 70+)	17
Obesity 2 – adult	0.88 (age 20-30); 0.83 (age 31-40); 0.79 (age 41-50); 0.77 (age 51-60); 0.76 (age 61-70); 0.74 (age 70+)	17
Obesity 3 - adult	0.84 (age 20-30); 0.82 (age 31-40); 0.75 (age 41-50); 0.73 (age 51-60); 0.71 (age 61-70); 0.69 (age 70+)	17
Disutilities		
Bariatric surgery	-0.22	14
Major complications from bariatric surgery	-0.36	14
Minor complications from bariatric surgery	-0.11	14

[a]We assumed adolescents undergoing treatment with anti-obesity drugs required 2 physician visits per year. For bariatric surgery, follow up costs were based on the following schedule: 5 visits in year 1, 3 visits in year 2 and 2 visits per year beyond year 2 for gastric bypass and 5 visits in year 1, 2 visits in year 2 and 1 visit per year beyond year 2 for sleeve gastrectomy[22]. Each follow-up visit costs US$134[16].

[b]Numbers denote treatment effects in each year post-surgery. Treatment effects for gastric bypass in year 4 were not available and were assumed as the average of the effects in years 3 and 5.

© 2023 Mital S and Nguyen HV. *JAMA Network Open.*

[c]Post-procedure complications could occur in the first 5 years after surgery. Data on post-procedure mortality were available for gastric bypass only. We assumed the risk of post-procedure mortality after sleeve gastrectomy was the same as after gastric bypass.

© 2023 Mital S and Nguyen HV. *JAMA Network Open.*

eFigure 1: Threshold time horizon[a]

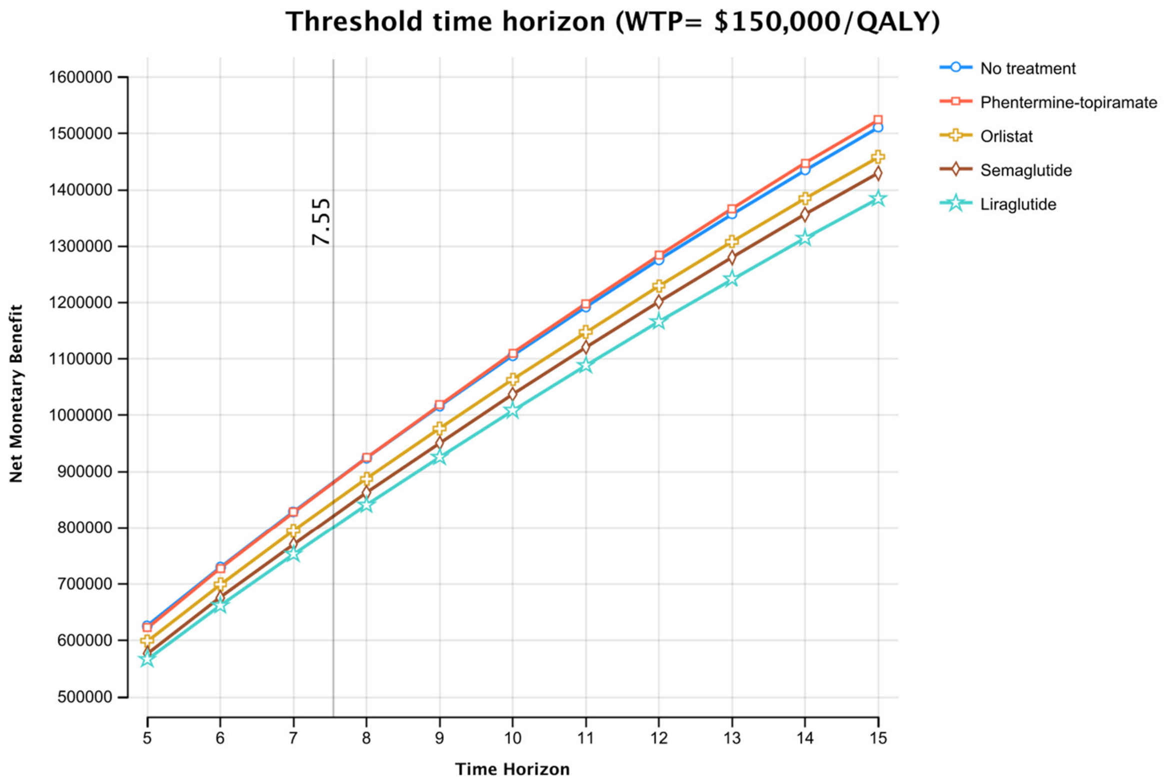

WTP: Willingness to Pay

[a]Net monetary benefit represents the monetary value of an intervention and is calculated by monetizing the incremental effectiveness (in QALYs) using the willingness to pay threshold of $150,000 per QALY and differencing the incremental cost of the intervention. At each time horizon, the strategy with the highest net monetary benefit is the best from the cost-effectiveness perspective.

© 2023 Mital S and Nguyen HV. *JAMA Network Open.*

eFigure 2: Threshold time to discontinuation[a]

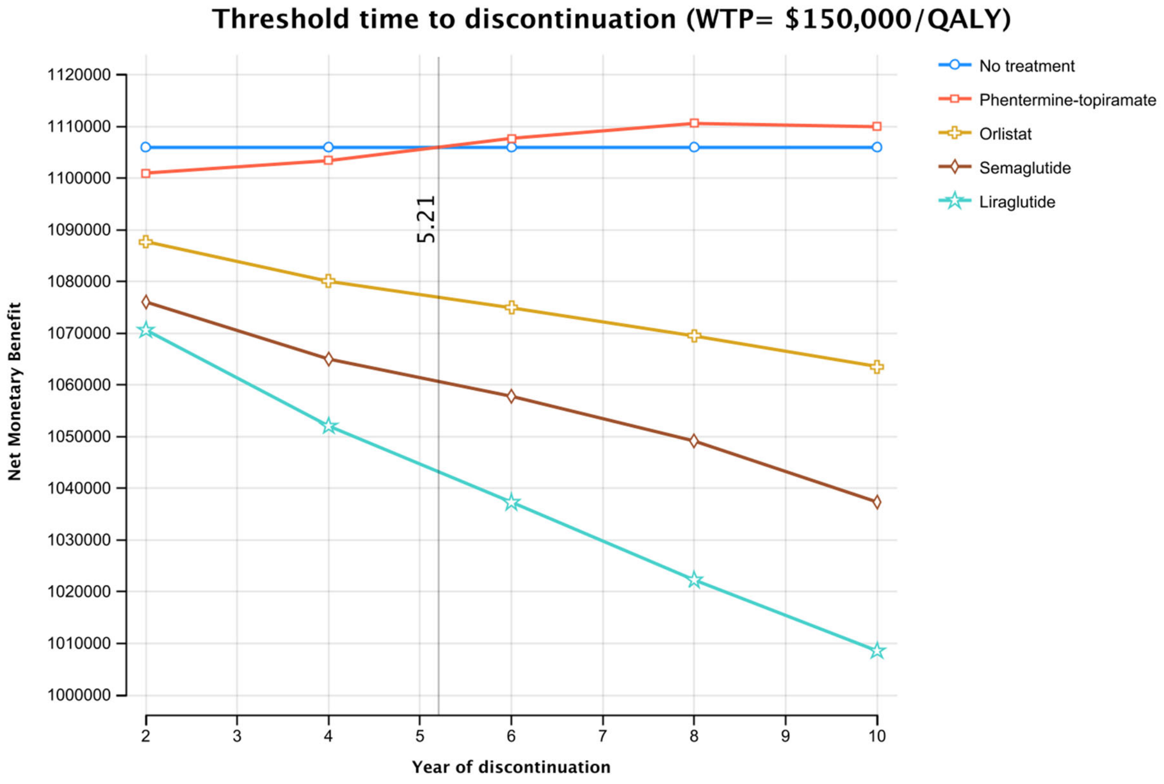

WTP: Willingness to Pay

[a]Net monetary benefit represents the monetary value of an intervention and is calculated by monetizing the incremental effectiveness (in QALYs) using the willingness to pay threshold of $150,000 per QALY and differencing the incremental cost of the intervention. At each year of discontinuation, the strategy with the highest net monetary benefit is the best from the cost-effectiveness perspective.

© 2023 Mital S and Nguyen HV. *JAMA Network Open.*

eFigure 3: Cost-effectiveness acceptability curve

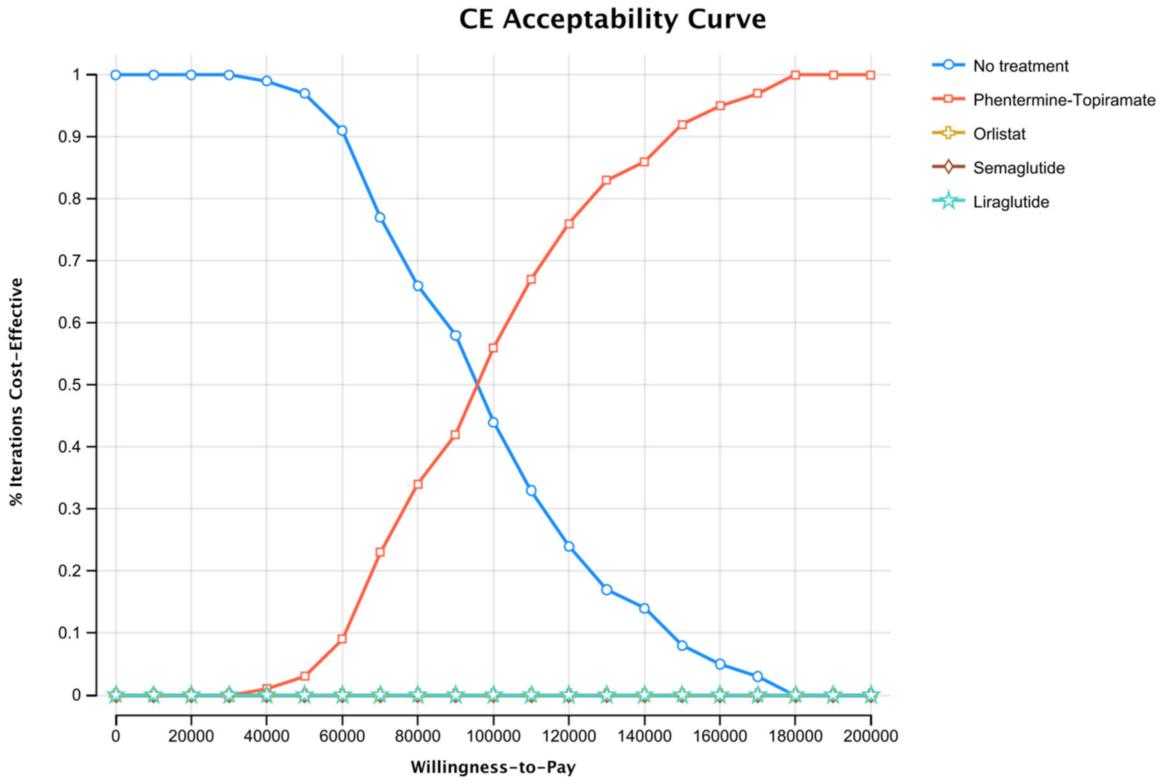

© 2023 Mital S and Nguyen HV. *JAMA Network Open.*

References

1. Kelly AS, Bensignor MO, Hsia DS, et al. Phentermine/topiramate for the treatment of adolescent obesity. *NEJM Evid*. 2022;1(6):EVIDoa2200014.

2. Chanoine JP, Hampl S, Jensen C, Boldrin M, Hauptman J. Effect of orlistat on weight and body composition in obese adolescents: a randomized controlled trial. *Jama*. 2005;293(23):2873-2883.

3. Weghuber D, Barrett T, Barrientos-Pérez M, et al. Once-weekly semaglutide in adolescents with obesity. *N Engl J Med*. 2022;387(24):2245-2257.

4. Kelly AS, Auerbach P, Barrientos-Perez M, et al. A randomized, controlled trial of liraglutide for adolescents with obesity. *N Engl J Med*. 2020;382(22):2117-2128.

5. Wilson DM, Abrams SH, Aye T, et al. Metformin extended release treatment of adolescent obesity: a 48-week randomized, double-blind, placebo-controlled trial with 48-week follow-up. *Arch Pediatr Adolesc Med*. 2010;164(2):116-123.

6. Inge TH, Courcoulas AP, Jenkins TM, et al. Weight loss and health status 3 years after bariatric surgery in adolescents. *N Engl J Med*. 2016;374(2):113-123.

7. Centers for Disease Control and Prevention. BMI for Children and Teens. Published March 21, 2023. Accessed April 29, 2023. https://www.cdc.gov/obesity/basics/childhood-defining.html

8. Levi J, Wang J, Venter F, Hill A. Estimated minimum prices and lowest available national prices for antiobesity medications: Improving affordability and access to treatment. *Obesity*. 2023;31(5):1270-1279. doi:10.1002/oby.23725

9. Wilding JP, Batterham RL, Davies M, et al. Weight regain and cardiometabolic effects after withdrawal of semaglutide: the STEP 1 trial extension. *Diabetes Obes Metab*. 2022;24(8):1553-1564.

10. McGinty SM, Osganian SK, Feldman HA, Milliren CE, Field AE, Richmond TK. BMI trajectories from birth to young adulthood. *Obesity*. 2018;26(6):1043-1049.

11. Inge TH, Courcoulas AP, Jenkins TM, et al. Five-year outcomes of gastric bypass in adolescents as compared with adults. *N Engl J Med*. 2019;380(22):2136-2145.

12. Veterans Affairs. Office of Procurement, Acquisition and Logistics (OPAL): Pharmaceutical Prices. Published 2023. Accessed April 29, 2023. https://www.va.gov/opal/nac/fss/pharmprices.asp

13. Sumarsono A, Sumarsono N, Das SR, Vaduganathan M, Agrawal D, Pandey A. Economic Burden Associated With Extended-Release vs Immediate-Release Drug Formulations Among Medicare Part D and Medicaid Beneficiaries. *JAMA Netw Open*. 2020;3(2):e200181. doi:10.1001/jamanetworkopen.2020.0181

© 2023 Mital S and Nguyen HV. *JAMA Network Open.*

14. Klebanoff MJ, Chhatwal J, Nudel JD, Corey KE, Kaplan LM, Hur C. Cost-effectiveness of bariatric surgery in adolescents with obesity. *JAMA Surg*. 2017;152(2):136-141.

15. Ward ZJ, Bleich SN, Long MW, Gortmaker SL. Association of body mass index with health care expenditures in the United States by age and sex. *PloS One*. 2021;16(3):e0247307.

16. Centers for Medicare and Medicaid Services. Gastroenterology Office Visit Costs. Published 2023. Accessed May 10, 2023. https://data.cms.gov/provider-data/search?fulltext=gast&theme=Physician%20office%20visit%20costs

17. Alsumali A, Eguale T, Bairdain S, Samnaliev M. Cost-effectiveness analysis of bariatric surgery for morbid obesity. *Obes Surg*. Published online 2018:1-12.

18. Borrell LN, Samuel L. Body Mass Index Categories and Mortality Risk in US Adults: The Effect of Overweight and Obesity on Advancing Death. *Am J Public Health*. 2014;104(3):512-519. doi:10.2105/AJPH.2013.301597

19. Inge TH, Zeller MH, Jenkins TM, et al. Perioperative outcomes of adolescents undergoing bariatric surgery: the Teen–Longitudinal Assessment of Bariatric Surgery (Teen-LABS) study. *JAMA Pediatr*. 2014;168(1):47-53.

20. Salminen P, Helmiö M, Ovaska J, et al. Effect of laparoscopic sleeve gastrectomy vs laparoscopic Roux-en-Y gastric bypass on weight loss at 5 years among patients with morbid obesity: the SLEEVEPASS randomized clinical trial. *Jama*. 2018;319(3):241-254.

21. Ward ZJ, Barrett JL, Cradock AL, et al. Childhood Obesity Intervention Cost-Effectiveness Study (CHOICES) Microsimulation Model Technical Documentation: Details on Model Parameters. Published 2023. https://choicesproject.org/wp-content/uploads/2023/03/CHOICES-Model-Technical-Documentation-v4.6.1.pdf

22. Clinical Practice Guidelines for the Support of the Bariatric Surgery Patient | ASMBS. American Society for Metabolic and Bariatric Surgery. Published March 1, 2013. Accessed March 27, 2019. https://asmbs.org/resources/clinical-practice-guidelines-for-the-perioperative-nutritional-metabolic-and-nonsurgical-support-of-the-bariatric-surgery-patient

© 2023 Mital S and Nguyen HV. *JAMA Network Open.*
