# Supplementary material for: Cost-Effectiveness of Antiobesity Drugs for Adolescents With Severe Obesity
Source: JAMA Netw Open. 2023 Oct 12;6(10):e2336400. doi: 10.1001/jamanetworkopen.2023.36400 (PMC10570871; doi:10.1001/jamanetworkopen.2023.36400)
Supplement: Supplement 2. — Data Sharing Statement [file jamanetwopen-e2336400-s002.pdf]

Data Sharing Statement

Mital. Cost-Effectiveness of Antiobesity Drugs for Adolescents With Severe Obesity. *JAMA Netw Open*. Published October 12, 2023. doi:10.1001/jamanetworkopen.2023.36400

Data
Data available: No

Additional Information
Explanation for why data not available: No individual patient data were used in this study. All data were sourced from the published literature and the sources are provided in the manuscript.
